# Continuous infusion of an agonist of the tumor necrosis factor receptor 2 in the spinal cord improves recovery after traumatic contusive injury

**DOI:** 10.1111/cns.13125

**Published:** 2019-04-02

**Authors:** Marcus J. Gerald, Valerie Bracchi‐Ricard, Jerome Ricard, Roman Fischer, Bharadwaj Nandakumar, Gary H. Blumenthal, Raushaun Williams, Roland E. Kontermann, Klaus Pfizenmaier, Karen A. Moxon, John R. Bethea

**Affiliations:** ^1^ Department of Biology Drexel University Philadelphia Pennsylvania; ^2^ Department of Biomedical Engineering Drexel University Philadelphia Pennsylvania; ^3^ Department of Biomedical Engineering University of California‐Davis Davis California; ^4^ Institute of Cell Biology and Immunology University of Stuttgart Stuttgart Germany; ^5^ Stuttgart Research Center Systems Biology University of Stuttgart Stuttgart Germany

## Abstract

**Aim:**

The activation of the TNFR2 receptor is beneficial in several pathologies of the central nervous system, and this study examines whether it can ameliorate the recovery process following spinal cord injury.

**Methods:**

EHD2‐sc‐mTNF_R2_, an agonist specific for TNFR2, was used to treat neurons exposed to high levels of glutamate in vitro. In vivo, it was infused directly to the spinal cord via osmotic pumps immediately after a contusion to the cord at the T9 level. Locomotion behavior was assessed for 6 weeks, and the tissue was analyzed (lesion size, RNA and protein expression, cell death) after injury. Somatosensory evoked potentials were also measured in response to hindlimb stimulation.

**Results:**

The activation of TNFR2 protected neurons from glutamate‐mediated excitotoxicity through the activation of phosphoinositide‐3 kinase gamma in vitro and improved the locomotion of animals following spinal cord injury. The extent of the injury was not affected by infusing EHD2‐sc‐mTNF_R2_, but higher levels of neurofilament H and 2′, 3′‐cyclic‐nucleotide 3′‐phosphodiesterase were observed 6 weeks after the injury. Finally, the activation of TNFR2 after injury increased the neural response recorded in the cortex following hindlimb stimulation.

**Conclusion:**

The activation of TNFR2 in the spinal cord following contusive injury leads to enhanced locomotion and better cortical responses to hindlimb stimulation.

## 1 INTRODUCTION

Spinal cord injury can lead to extensive damage to the nervous tissue resulting in serious deficits in sensorimotor functions. Neuronal cell death and glial cell death occur either rapidly following the initial mechanical damage, or during the following secondary phase that can last for weeks and which involves a neuroinflammatory response to the injury.^1^

Resident astrocytes and microglia, as well as immune cells infiltrating the spinal cord after the injury, mediate the neuroinflammatory response and secrete several pro‐inflammatory cytokines, including tumor necrosis factor (TNF).

Tumor necrosis factor is essential in triggering healing mechanisms but can initiate both cell death or survival pathways depending on the pathophysiological conditions. It is produced under two biologically active forms, a soluble one (solTNF) resulting from the regulated cleavage of the extracellular domain of the transmembrane form (tmTNF). SolTNF signals specifically through the ubiquitously expressed receptor TNFR1, while tmTNF is able to bind both TNFR1 and TNFR2.^2^ Unlike TNFR1, TNFR2 is expressed by a restricted number of cells (immune cells, endothelial cells, as well as neurons, astrocytes, and oligodendrocytes in the central nervous system (CNS)) and lacks a death domain, therefore triggering mostly prosurvival pathways by activating phosphoinositide‐3 kinase (PI3K) and NF‐κB.^3^, ^4^

Although the inhibition of TNF (both soluble and transmembrane forms) in clinical trials, aimed at treating patients suffering from multiple sclerosis, surprisingly exacerbated the disease,^5^, ^6^ there are many examples of favorable outcomes when only the signals elicited by solTNF are inhibited while TNFR2 signaling is maintained, demonstrating the beneficial effects of tmTNF in CNS pathologies. Studies in experimental autoimmune encephalomyelitis (EAE), a murine model of MS, describe a more severe pathology in animals lacking TNFR2 expression.^7^ Moreover, selective inhibition of solTNF results in an improved clinical outcome, whereas inhibitors blocking all TNF signaling did not have any noticeable effect on the disease course.^8^ TNFR2 activity also promotes remyelination and oligodendrocyte precursor cells (OPCs) proliferation after cuprizone‐induced demyelination,^9^ the proliferation of OPCs being mediated by CXCL12 released from activated astrocytes through TNFR2 signaling.^10^ TNFR2 can also protect OPCs from oxidative stress.^11^

In vitro studies have shed some light on the protective effects and mechanisms mediated by TNFR2: Inhibition of TNFR2 in neuronal cells enhanced the susceptibility of these cells to hypoxic insults or beta‐amyloid toxicity,^12^ whereas oligodendrocytes matured in response to leukemia inhibitory factor (LIF) secreted by astrocytes in response to TNFR2 stimulation.^13^

In the case of spinal cord injury, solTNF is upregulated and contributes to apoptosis,^14^ an increased trafficking of AMPA receptors at the plasma membrane triggered by TNF exacerbating the process.^15^ The lack of either both TNF forms^16^ or solTNF only^17^ does not provide any protection or improvement following injury; however, short‐term inhibition of solTNF (3 days) signaling is beneficial.^18^ After having reported a decreased expression of TNFR2 in the latter study after spinal cord injury and having observed effects of TNFR2 signaling in neurons and OPCs in vitro that one might consider beneficial after a traumatic insult to the cord, we wondered whether enhancing TNFR2 signaling activity through stimulation with a specific agonist might enhance recovery. Our data show that in vitro TNFR2 stimulation protects neurons against glutamate‐induced cytotoxicity. In vivo, following spinal cord injury, stimulation of TNFR2 helps improving locomotion, as well as remodeling neuronal circuitry.

## 2 MATERIALS AND METHODS

### 2.1 Cell culture

The EHD2‐sc‐mTNF_R2_ agonist was described previously.^19^ E18 rat cortical neurons purchased from Neurons‐R‐Us (Mahoney Institute of Neurological Sciences, University of Pennsylvania) were cultured in Neurobasal medium (Thermo Fisher) supplemented with B27 on poly‐D‐lysine/laminin‐coated 96‐well plates (10^5^ cells/well) for 7 days before treatments.

### 2.2 Cell death assay

Neurons were pretreated with the TNFR2‐specific agonist EHD2‐sc‐mTNF_R2_ (1 µg/mL) before stimulation with glutamate (300 µmol/L) for one hour. A66 (0.2 µmol/L), AS 605240 (32 nmol/L), and SB203580 (5 µmol/L) (Tocris) (respectively, inhibitors of the PI3K catalytic subunits p110α and p110γ, and p38 MAPK) were added at the same time as the EHD2‐sc‐TNF_R2_ agonist.

Cell death was assessed by staining with the HCS LIVE/DEAD Green Kit (Thermo Fisher). Briefly, the neurons were incubated with the Image‐iT DEAD Green solution for 30 minutes. The cells were then washed with phosphate‐buffered saline (PBS) three times and fixed using 16% paraformaldehyde (PFA) for 15 minutes, and all nuclei were counterstained with Hoechst 33342. PBS was then added to each well before imaging on a Zeiss Axio Observer wide field microscope using a 5× objective.

### 2.3 Mice and spinal cord injury

All animal experiments followed NIH and Drexel University IACUC guidelines for the use of laboratory animals.

Adult female C57Bl/6 mice (3 months old) were purchased from Jackson Laboratory and were acclimated for at least 1 week before undergoing surgery as previously described.^18^ Briefly, following anesthesia with ketamine (100 mg/kg) and xylazine (10 mg/kg), a laminectomy was performed at thoracic level T9 and the exposed spinal cord was contused using the Infinite Horizon impactor (Precision Systems and Instrumentation, LLC) with a desired force of 65 kDynes. Following injury, muscles of the back, as well as the skin, were sutured. After injury, lactated Ringer's solution was administered subcutaneously twice daily for at least 7 days to prevent dehydration, buprenorphine (0.1 mg/kg) twice a day for 2 days for pain relief and gentamicin (80 mg/kg) once a day for 7 days as a preventive measure against bladder infection. Bladders were expressed twice daily until normal function was recovered. Data related to the injuries are reported in Table S1.

### 2.4 Drug administration

Micro‐osmotic pumps connected to brain infusion kits III (ALZET), affixed to the vertebrae with the cannula placed above the lesion immediately following injury, were used to deliver EHD2‐sc‐mTNF_R2_. The concentrations were adjusted according to the experiment conducted to maintain daily delivery of similar amounts: 10 mg/mL for 28 days of delivery (model 1004:0.11 µL/h; BMS, 6 weeks of protein analysis), 4.4 mg/mL for 14 days of delivery (model 1002:0.25 µL/h; 2 weeks of protein analysis, electrophysiology), and 1.1 mg/mL for 3 days of delivery (model 1003D: 1 µL/h; RNA analysis).

### 2.5 Electrophysiology and locomotion behavior

The animals' locomotion during the recovery period was assessed using the BMS scoring system developed by Basso et al.^20^ The animals were tested at day 1 after injury (the animals not displaying paralysis were excluded from the study) and then once a week for six consecutive weeks.

#### 2.5.1 Electrophysiology

Two weeks after injury, animals were anesthetized with 1.5 g/kg of urethane via intraperitoneal injection and were secured in a stereotaxic frame (Stoelting Co., Wood Dale, IL). Body temperature was kept constant (36.5°C) using a temperature feedback‐controlled heating pad, and heart rate and partial pressure O_2_ were constantly monitored (Kent Scientific Co., Torrington, CT). An incision was made along the midline of the scalp, and the skin was retracted. A craniotomy was performed on the right side of the skull exposing the hindlimb sensory cortex. A 32‐channel, single shank recording electrode (A1x32, NeuroNexus, Ann Arbor, MI) was mounted to a stereotaxic manipulator and positioned over the hindlimb sensory cortex (0.2 mm caudal and 2 mm lateral to bregma), perpendicular to the surface of the brain. A cortical screw was inserted into the left side of the skull such that it was placed in contact with cerebral spinal fluid and was used as the electrode's reference. The electrode was advanced into the cortex to a depth of 1.65 mm at a rate of approximately 50 µm per minute, while neural signals were amplified (192X), band‐pass filtered (0.1 Hz‐7.5 kHz), and monitored on an oscilloscope and through audio speakers. Analogue neural signals were acquired and converted to a digital signal at a sampling rate of 20.0 kS/s and 16‐bit quantization (Intan Technologies, Los Angeles, CA).

#### 2.5.2 Peripheral electrical stimulation

Bipolar stimulating electrodes were inserted subcutaneously into the ankle of the hindlimb contralateral to the recording site (Figure S1A). For each animal, electrical stimulation, consisting of 100 pulses at 0.5 Hz with a 1.0 ms pulse duration at 1.0 mA of current, was applied as the neural response was recorded.

#### 2.5.3 Evoked responses

Local field potentials (LFPs) were obtained from each recording site on the electrode by applying a low‐pass filter (200 Hz, Butterworth order 5, Zero lag) to the raw signal. One second windows of neural data centered around each stimulation time point were averaged across all stimulation trials in a single recording to generate a sensory evoked potential (SEP). A representative channel from the supragranular (150 µm depth), granular (500 µm depth), and infragranular (800 µm depth) layers of the cortex was selected for further analysis. We then evaluated whether there was a cortical response to the stimulation. For each layer, the SEP was considered responsive whether its peak amplitude exceeded a response threshold equal to the mean plus 3 standard deviations of the background activity. For each responsive SEP, the amplitude was calculated as the difference between the SEP peak amplitude and the response threshold. If the SEP peak amplitude did not exceed the response threshold, it was considered not responsive, and the value of the SEP amplitude was set to zero. The SEP latency was evaluated for responsive SEPs and was defined as the time of the SEP peak amplitude relative to the stimulation. The proportion of positive responses, and the SEP amplitudes and latencies for positive responses were compared across groups separately for the supragranular, granular, and infragranular layers of the hindlimb somatosensory cortex.

To ensure that we were accurately placing the recording sites at the proper depth, current source density analysis was used to confirm electrode placement. In naïve animals, CSD profiles of responses to hindlimb stimulation were obtained. The strongest current sink in the naïve animals corresponded to a depth of 500 microns and was estimated to be the granular layer (Figure S1B,C).^21^, ^22^, ^23^, ^24^

### 2.6 Spinal cord tissue analysis

Measurement of lesion volume and immunohistochemistry: The animals were perfused transcardially with phosphate‐buffered saline followed by ice‐cold 4% paraformaldehyde (PFA). The tissue was postfixed for 4 hours in PFA before transfer to 25% sucrose overnight. The cords were embedded and frozen in OCT (Tissue‐Tek) before sectioning with a cryostat. Thirty micrometre transversal sections were stained using rabbit anti‐GFAP antibodies (1:1000, DAKO) to identify the edge of the lesion. Four sections 300 µm apart were used, and the volume of the lesion was estimated using the Stereology Module of Slidebook6 software. TUNEL staining was performed to identify cell death using the ApopTag staining kit (EMD Millipore).

### 2.7 Protein expression and RNA analysis

0.5 cm long segments of spinal cord tissue centered on the lesion site were collected at various time points after injury following PBS perfusion of the animals (different animals from BMS group) and flash‐frozen on dry ice. For proteins, the tissues were lysed in RIPA buffer (10 mmol/L sodium phosphate pH 7.2, 150 mmol/L NaCl, 2 mmol/L EDTA, 1% sodium deoxycholate, 1% NP‐40, 0.1% sodium dodecyl sulfate) supplemented with protease inhibitors (Pierce tablets) and phosphatase inhibitors (BioVision). Proteins were resolved on SDS‐PAGE gels before being transferred onto nitrocellulose membranes using the Trans‐Blot^® ^Turbo™ system (Bio‐Rad). Antibodies for the Western blot detection included rabbit anti‐CNPase (1:5000; Cell signaling), mouse anti‐NF200 (1:5000; Sigma), rabbit anti‐PI3Kγ (1:500; Santa Cruz), and mouse anti‐β‐tubulin (1:60 000; Sigma).

RNAs were extracted using TRIzol (Thermo Fisher) following the manufacturer's instructions. RT‐PCR was performed with the Omniscript RT kit (Qiagen) followed by qPCRs performed using the Rotor‐Gene SYBR^®^ Green PCR Kit, and the Rotor‐Gene Q real‐time PCR cycler (Qiagen). Each gene expression levels were determined using a standard curve for that gene, and the data were normalized to β‐actin expression levels. The primers used are listed in the Table S2.

### 2.8 Statistical analysis

Comparisons between groups were performed using one‐way analysis of variance (ANOVA) followed by post hoc Tukey's test. If only two groups, a two‐tailed *t* test was used.

#### 2.8.1 Behavior

A two‐way ANOVA was performed.

#### 2.8.2 Electrophysiology

The proportion of responses was compared across groups for each layer separately using the chi‐squared test with the Fisher's exact test post hoc. Changes in SEP amplitude and latency were not normally distributed. Kruskal‐Wallis test was used to evaluate statistical differences in SEP amplitude and latency between groups, separately for each layer. Post hoc Mann‐Whitney *U* test assessed differences between PBS controls and treated animals if the Kruskal‐Wallis was significant. All results were considered statistically significant at *P* < 0.05.

## 3 RESULTS

### 3.1 Activation of TNFR2 protects neurons against glutamate‐induced cell death

**Figure 1:**
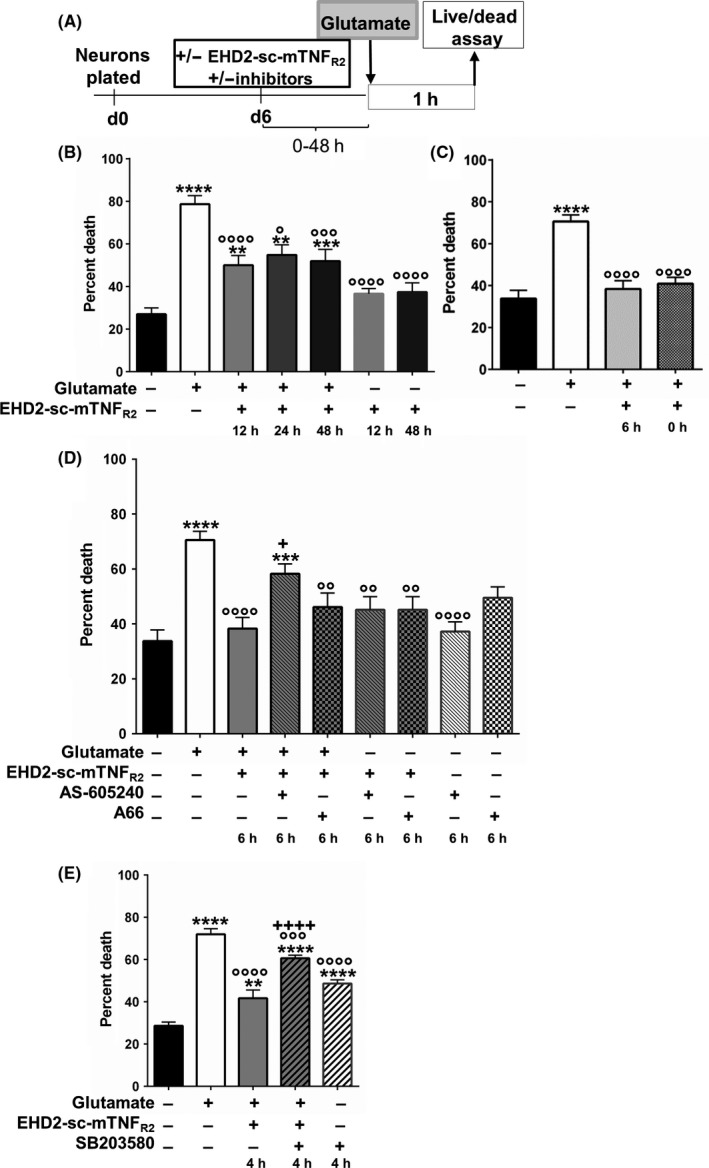
Effect of TNFR2 activation on glutamate‐induced neuronal cell death. A, Timeline of the experiment. B, Effect of pretreatment with EHD2‐sc‐mTNF_R2 _on glutamate‐induced neuronal cell death. Cells were pretreated for 12 h, 24 h, or 48 h prior to addition of 300 μmol/L of glutamate. C, Effect of treatment with EHD2‐sc‐mTNF_R2 _at time (0h) or 6 h prior the addition of glutamate on cell death. D, Role of PI3Kγ in the protection provided by EHD2‐sc‐mTNF_R2 _on glutamate‐induced neuronal cell death. Cells were pretreated for 6 h with EHD2‐sc‐mTNF_R2_ alone or in combination with either A66 (PI3K/p110α inhibitor) or AS 605240 (PI3K/p110γ inhibitor). E, Role of p38 MAPK in glutamate‐induced cell death. Cells were pretreated for 4 h with either EHD2‐sc‐mTNF_R2_ alone or with SB203580 (p38 inhibitor). All bar graphs represent the mean ± SEM of eight separate samples for each condition. ****P < 0.0001; ***P < 0.001; **P < 0.01; *P < 0.05 (compared to untreated controls). ºººº*P* < 0.0001; ººº*P* < 0.001; ºº*P* < 0.01; º*P* < 0.05 (compared to glutamate controls). +*P* < 0.05, ++++*P* < 0.0001 (compared to glutamate/EHD2‐sc‐mTNF_R2_)

Given the body of evidence describing the neuroprotective role of TNFR2, we studied how neurons would respond to stimulation with a specific agonist for this receptor. A previous study demonstrated a protective effect against glutamate excitotoxicity after antibody‐mediated pre‐activation of TNFR2 through the activation of PI3 kinase.^25^ We reexamined these experiments with the specific agonist EHD2‐sc‐mTNF_R2 _(Figure 1A). A short pretreatment (6 hours) with TNFR2 agonist before treatment with glutamate decreased noticeably the amount of cells dying compared to cells pretreated for a longer time (12, 24 or 48 hours) (Figure 1B, C). Interestingly, stimulation of TNFR2 performed simultaneously with exposure to glutamate was also able to prevent the neurons from dying as effectively as a treatment with the agonist initiated 6 hours earlier (Figure 1C). Using inhibitors targeting different classes of PI3K catalytic subunits, we observed that p110γ was the protein mediating the signal provided by TNFR2 activation (Figure 1D), as treatment with AS605240 abrogated the protective effect elicited by EHD2‐sc‐mTNF_R2_. A similar experiment using the p110α inhibitor A66 did not block the protective effect triggered by TNFR2 stimulation. We then examined the potential involvement of p38 MAPK on the pathway activated by EHD2‐sc‐mTNF_R2_ as this kinase has been implicated in PI3Kγ signaling^26^ and observed that its inhibition abolished the protection from glutamate excitotoxicity provided by activation of TNFR2 (Figure 1E).

### 3.2 Effect of EHD2‐sc‐mTNF_R2_ on recovery following spinal cord injury

**Figure 2:**
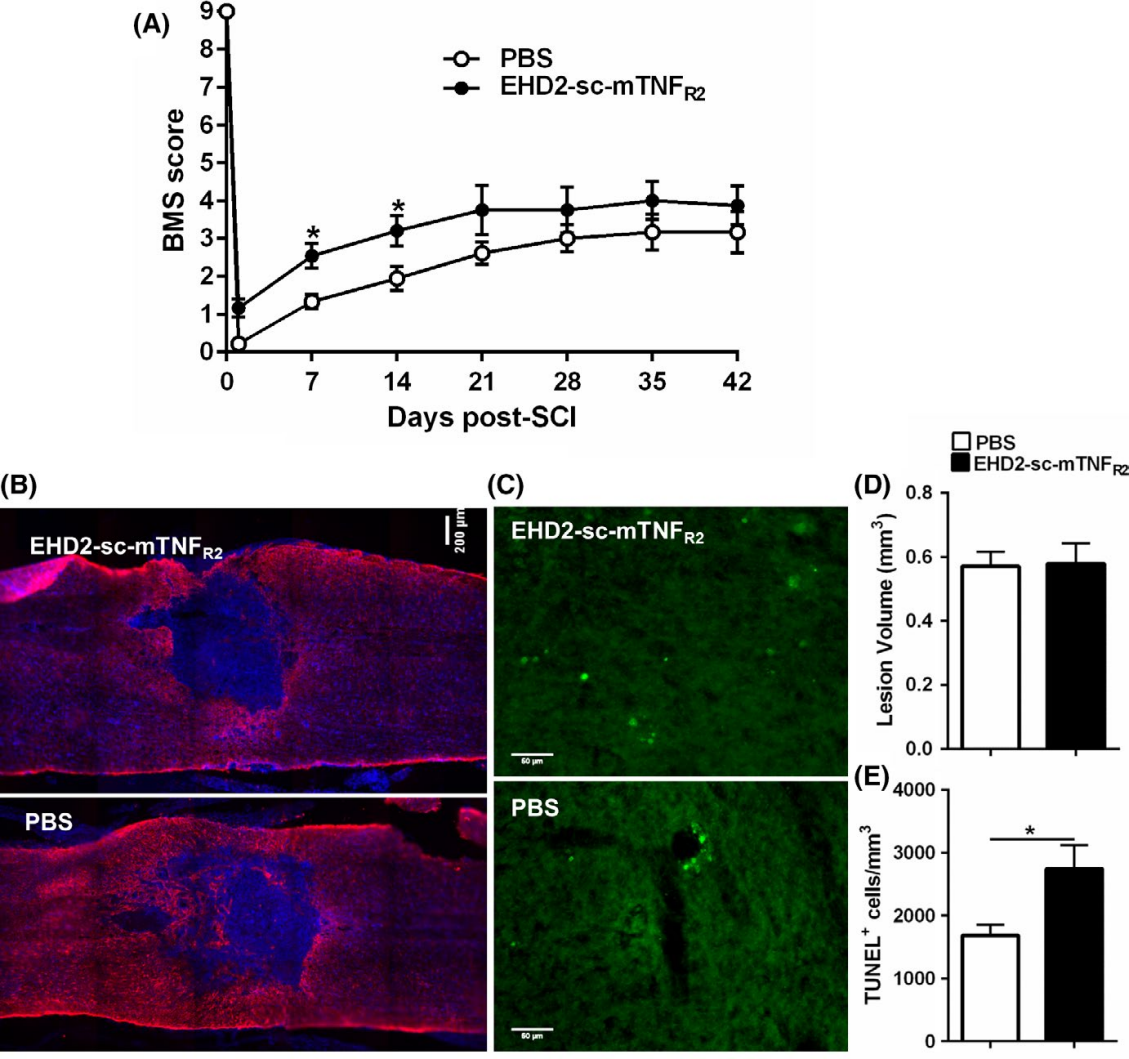
Effect of EHD2‐sc‐mTNF_R2_ on locomotion after spinal cord injury. A, Effect of TNFR2 agonist on locomotion recovery (assessed by BMS scoring 1 d after injury and weekly thereafter for 6 weeks). *P < 0.05. B, Representative longitudinal sections of spinal cords showing the lesion 2 wk after contusive injury. Blue: Hoechst; red: staining for GFAP. C, Representative images of the TUNEL staining in spinal cords 2 wk after injury. D, Quantification of lesion size. E, Quantification of TUNEL‐positive cells detected in the spinal cord 2 weeks following injury. Bar graphs represent the mean ± SEM of n = 6 PBS and n = 5 EHD2‐sc‐mTNF_R2_* *P* < 0.05

**Figure 3:**
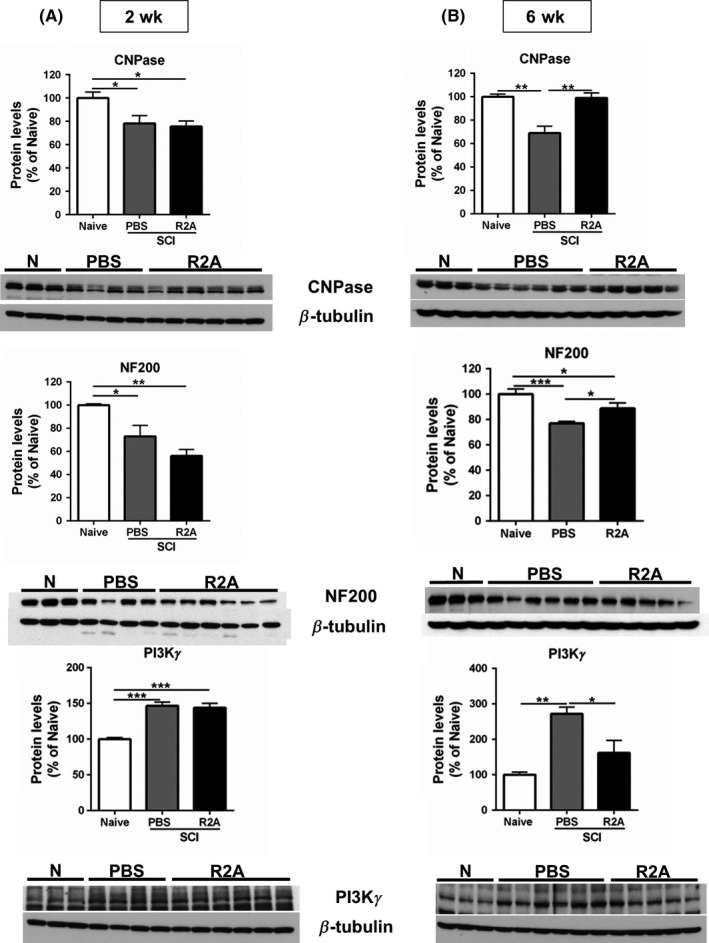
Expression levels of proteins around the lesion site following spinal cord injury. A, Expression of CNPase, neurofilament H (NF200), and PI3Kγ 2 weeks following injury. Bar graphs represent the mean ± SEM of n = 3 naïve (N), n = 4 PBS, and n = 6 R2A. ***P < 0.001; **P < 0.01; *P < 0.05. B, CNPase, NF200, and PI3Kγ protein expression, 6 weeks following injury. Last sample in the NF200 blot was an outlier as determined by the Grubb's method and was excluded from the bar graph. Bar graphs represent the mean ± SEM of n = 3 naïve, n = 6 PBS, and n = 5 R2A ***P < 0.001; **P < 0.01; *P < 0.05. PBS: spinal cord injured controls infused with PBS; R2A: spinal cord injured animals infused with EHD2‐sc‐mTNF_R2_

Since activating TNFR2 with a specific agonist can have effects on two cell types (neurons and oligodendrocytes) that would be considered positive in order to mitigate the consequences of spinal cord trauma, we then analyzed whether infusion of the agonist directly into the spinal cord tissue after injury in mice could reduce the extent of damage and/or promote repair. EHD2‐sc‐mTNF_R2_ was infused continuously over a month and locomotion assessed weekly with the BMS test. We observed that stimulation of TNFR2 in the cord tissue improved the locomotion of the animals (Figure 2A). A significant improvement was observed at 1 and 2 weeks after injury, with greater BMS scores measured for the entire testing period. The improved locomotion did not result from extensive sparing of cord tissue as the lesion size was similar regardless of treatment (Figure 2B, D), even with greater numbers of TUNEL‐positive cells detected, indicating increased cell death (Figure 2C, E). The levels of several proteins that could provide insight about the pathological changes taking place in the spinal cord were examined. No changes in any protein analyzed were observed before 6 weeks after injury when significantly higher levels of neurofilament H (NF200) and 2′, 3′‐cyclic‐nucleotide 3′‐phosphodiesterase (CNPase) were detected (Figure 3A, B). Several proteins studied, especially proteins that are part of the myelin sheath such as myelin basic protein (MBP) or proteolipid protein (PLP), did not appear to be affected by our treatment. We also did not observe any changes in the expression of glial fibrillary acidic protein (GFAP) or Iba‐1, a marker of activated microglia (not shown). Interestingly, although we identified PI3Kγ as a mediator of the neuroprotection pathway activated by TNFR2, the levels of that protein were significantly lower after treatment with the TNFR2 agonist 6 weeks following the contusion compared to PBS treatment (Figure 3B).

### 3.3 TNFR2 activation does not modulate cytokine expression in the acute phase

**Figure 4:**
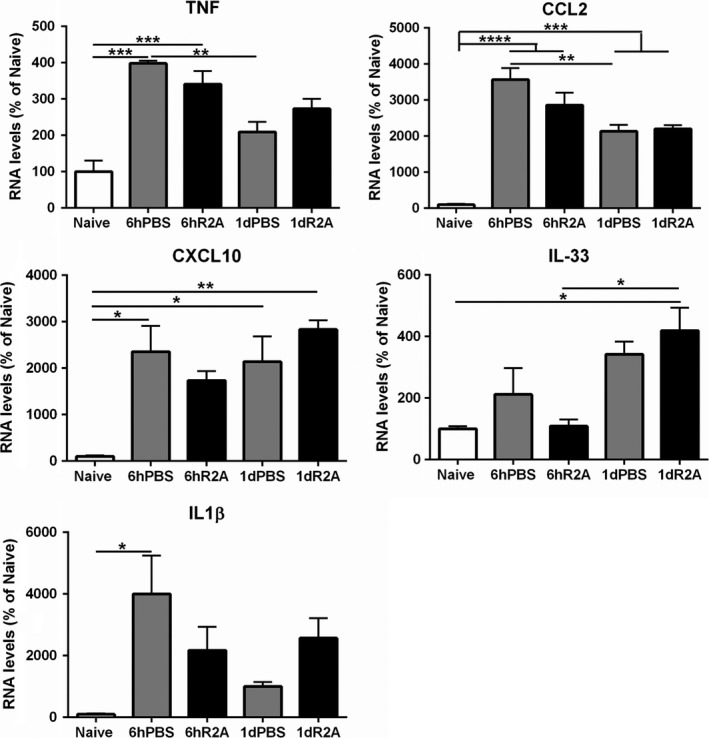
Acute response of genes linked to the inflammatory response after spinal cord injury. The RNA levels were measured 6 h and 1 d after injury by quantitative real‐time PCR. Data for each gene were normalized to β‐actin and expressed as a percentage of the naïve uninjured control. Bar graphs represent the mean ± SEM of n = 3/group/time point *P < 0.05, **P < 0.01, ***P < 0.001, ****P < 0.0001

TNFR2 stimulation has been associated with a decrease in immunological response in some models, which led us to consider whether the modulation of early inflammatory signaling could be affected by the TNFR2 agonist. The levels of some gene transcripts involved in inflammatory pathways were analyzed 6h and 1d after contusion in the spinal cord, but we did not observe any significant changes triggered by TNFR2 agonist in the levels of TNF, IL‐1β, IL‐33, CCL2, and CXCL10 compared to PBS (Figure 4 ).

**Figure 5:**
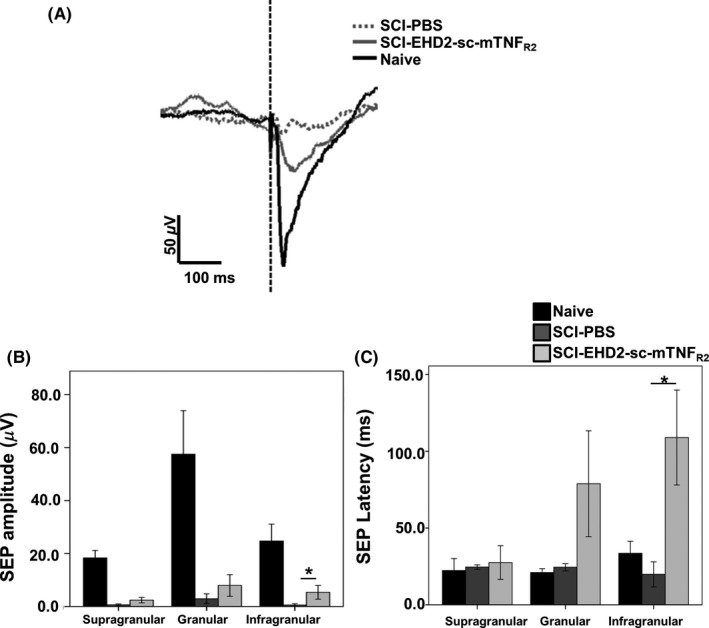
Effect of TNFR2 activation in the injured spinal cord on somatosensory evoked responses (SEP) in the hindlimb cortex. A, Representative trace of SEP recorded from the infragranular layer following hindlimb stimulation. B, Quantification of the amplitude of SEP recorded from three cortical layers. Injured animals have smaller amplitudes compared to naïve ones, but stimulation of TNFR2 increases significantly the amplitude of SEP recorded in the infragranular layer. *P < 0.05. C, Quantification of the latency of SEP recorded from three cortical layers. The animals treated with EHD2‐sc‐mTNF_R2_ show a significantly greater latency for the SEP recorded in the infragranular layer. *P < 0.05

### 3.4 Enhanced cortical response activation after spinal cord injury following TNFR2

Finally, a mounting body of evidence points to axon sprouting and cortical reorganization that can contribute to the recovery following spinal cord injury, even in the case of a complete transection.^27^ To determine whether the effect of the TNFR2 agonist could affect the cortical response to sensory stimuli, the response to hindlimb stimulation was compared across groups. As expected, the injury resulted in a sharp decrease in the amplitude of the SEP for all three layers (Figure 5B) (supragranular: H(2) = 18.636, *P* < 0.05; granular: H(2) = 14.568, *P* < 0.05; and infragranular: H(2) = 16.311, *P* < 0.01). However, treatment with EDH2‐sc‐mTNF_R2_ resulted in a significantly greater amplitude of the SEP for the infragranular layer compared to the animals infused with PBS only (Figure 5A, B) (U (1) = 30, Z = 2.015, *P* < 0.05, Cohen's *R* = 0.43). Moreover, the SEP latency was significantly longer in the infragranular layer following infusion with the TNFR2 agonist (Figure 5C) (U (1) = 0, z = −2, *P* < 0.05, Cohen's *R* = −0.7). Given the layer specificity of the effect on amplitude and latency, it is worth noting that, in the infragranular layer, there was a trend for more positive responders in the animals infused with the TNFR2 agonist compared to the ones infused with PBS (Fisher's test, *P* = 0.063). Together, these data suggest that, in animals treated with EHD2‐sc‐mTNF_R2_, sprouting at the level of the lesion may increase the probability of an evoked response and those responses will have a larger amplitude and longer latency than in animals where TNFR2 was not stimulated.

## 4 DISCUSSION

We report observing several potentially beneficial effects promoted by the specific activation of TNFR2 following spinal cord injury that could contribute to a positive effect on the recovery process. The infusion of the agonist EHD2‐sc‐mTNF_R2_ over a month after contusive thoracic spinal cord injury allowed the animals to exhibit better locomotion as assessed with the BMS test. Early activation of TNFR2 did not reduce inflammation, as could have been suspected from recent work demonstrating that the activation of that receptor in microglia curbs the inflammatory response in experimental autoimmune encephalomyelitis.^28^ TNFR2 activation may also have a direct protective effect on neurons in the cord as it is able to preserve them in vitro from an exposure to excessive glutamate concentrations, which are known to increase following spinal cord injury and cause excitotoxic cell death.^29^, ^30^ We present evidence here that the activation of TNFR2 does not need to precede the glutamate insult as previously thought,^25^ but rather that it can occur simultaneously and that it is mediated by PI3Kγ. We observed a noticeably stronger protective effect with shorter times between TNFR2 stimulation and glutamate exposure (0 or 6 hours, compared to 12 hours and up), suggesting that the signaling may be downregulated to levels inadequate to provide complete protection from glutamate excitotoxicity if TNFR2 gets activated too early. Surprisingly, while PI3Kγ was increased at 2 weeks after injury, there was no difference between EHD2‐sc‐mTNFR2 and PBS treated groups. Furthermore, the levels of PI3Kγ were significantly lower at 6 weeks in the group treated with the TNFR2 agonist compared to PBS. However, any neuroprotective effect mediated by PI3Kγ would most likely occur early after injury, within hours or a few days at most. Besides its protective effect from glutamate‐induced excitotoxicity, PI3Kγ contributes to neuroinflammation,^31^ and a decreased expression because of activation of TNFR2 could prevent inflammation to be sustained at levels that could prove harmful over long periods of time. Despite the positive effects observed in vitro on neurons in our study and OPCs^9^, ^10^ following TNFR2 stimulation, we could have expected some tissue sparing to be the result of EHD2‐sc‐mTNF_R2_ infusion, yet we did not observe substantive changes in the size of the lesion. One potential effect of TNFR2 stimulation on neurons that could result in improved behavior in the absence of increased tissue sparing may be enhanced neural circuit reorganization as suggested by our electrophysiological recordings. Infusion with EHD2‐sc‐mTNF_R2_ increased the probability of an evoked response having a larger amplitude and a longer latency following hindlimb stimulation, which may be explained by enhanced connectivity due to sprouting and an increased number of synapses for the signal to cross. Cortical reorganization following spinal cord injury can be associated with increased pain.^32^ However, TNFR2 activation can stimulate the expansion of the population of T‐regulatory cells,^33^, ^34^ which can alleviate pain in experimental autoimmune encephalomyelitis.^35^ T‐regulatory cells are also neuroprotective and can favor myelination in the CNS.^36^, ^37^ Our study yielded one counterintuitive result when we observed an increased number of cells dying as a result of TNFR2 activation in the cord. Such an increase could be expected to correlate with greater tissue damage and poorer behavioral outcome, yet we observed the opposite on both counts. We were not able to accurately characterize the phenotype of the TUNEL‐positive cells as specific proteins are lost during apoptosis. Our in vitro data would tend to suggest that the cells dying in greater numbers may not include neurons, and possibly oligodendrocytes. The survival of immune cells, such as T cells and macrophages, has been shown to be sensitive to TNFR2 stimulation.^38^, ^39^ These cells are among many that infiltrate the cord following injury and that can both participate to the healing or the exacerbation of the damage caused by injury,^40^ and their elimination, after performing beneficial duties such as phagocytosing debris in the case of macrophages, could prove beneficial.

Our data describe a positive effect triggered by stimulation of the TNFR2 receptor in the spinal cord following a contusive injury that may have several origins. The most promising and possibly most important one is the effect on neurons, where these cells could be spared from secondary injury processes in greater numbers and be stimulated to sprout and establish more connections to bypass the site of injury.

## DISCLOSURE

The authors declare no conflicting financial interest.

## AUTHOR CONTRIBUTION

MJG, VB‐R. JR, and JRB designed the research study; RF, REK, and KP designed and produced the TNFR2 agonist; MJG, VB‐R., JR, RF, BN, GHB, and RW conducted experiments and acquired data; all authors analyzed data; VB‐R., JR, and JRB wrote the manuscript; all authors contributed discussion to the manuscript.
