# Supplementary figures and images for: Continuous infusion of an agonist of the tumor necrosis factor receptor 2 in the spinal cord improves recovery after traumatic contusive injury
Source: CNS Neurosci Ther. 2019 Apr 2;25(8):884–93. doi: 10.1111/cns.13125 (PMC6630008; doi:10.1111/cns.13125)

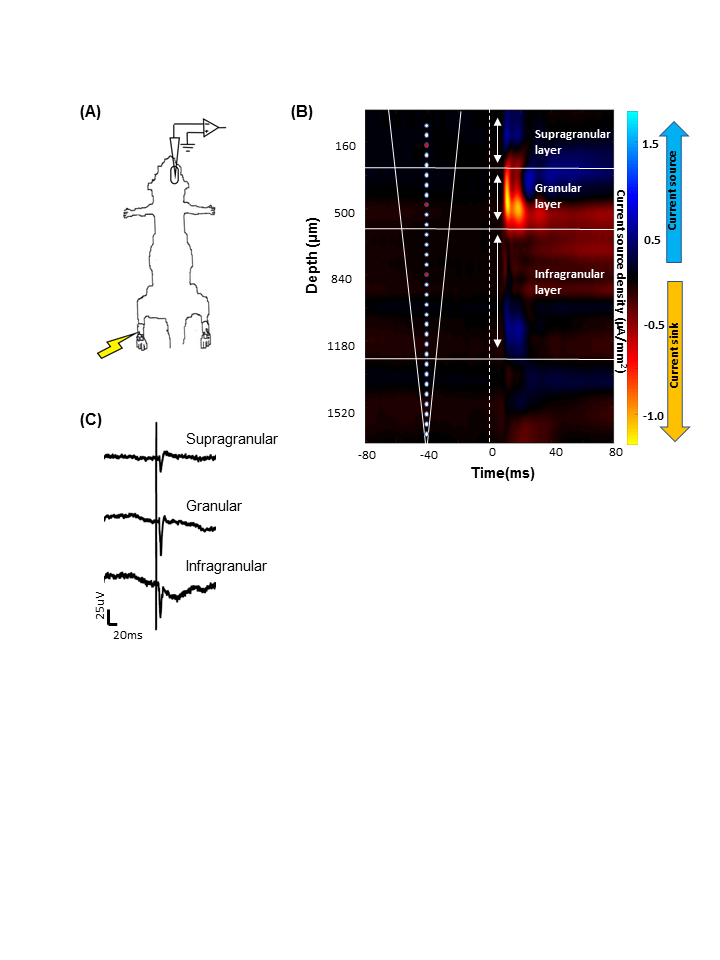

Supplement: Supplementary file 1 [file CNS-25-884-s001.tif]
