# Supplementary material for: Continuous infusion of an agonist of the tumor necrosis factor receptor 2 in the spinal cord improves recovery after traumatic contusive injury
Source: CNS Neurosci Ther. 2019 Apr 2;25(8):884–93. doi: 10.1111/cns.13125 (PMC6630008; doi:10.1111/cns.13125)
Supplement: Supplementary file 2 [file CNS-25-884-s002.docx]

| Experiment | group | sample  size | force  (kdynes ± SD) | displacement  (µm ± SD) | animals excluded * |
| --- | --- | --- | --- | --- | --- |
| BMS test | PBS | 9 | 66.3 ± 1.4 | 652.1 ± 127.8 | 3 |
|  | TNR2 agonist | 12 | 66.7 ± 2.09 | 679.9 ± 105.6 | 1 |
|  | naive | 8 | n/a | n/a | n/a |
| electrophysiology | PBS | 11 | 70.7 ± 9.9 | 546.3 ± 94.6 | 1 |
|  | TNR2 agonist | 10 | 68.2 ± 2.6 | 533 ± 64.0 |  |
|  | naive | 3 | n/a | n/a | n/a |
|  |  |  |  |  |  |
| RNA analysis | PBS | 6 | 68.3 ± 2.5 | 569.7 ± 183. 6 | 0 |
|  |  |  |  |  |  |
|  | TNFR2 | 6 | 67.2 ± 2.2 | 563.8 ± 111.1 | 0 |
|  | agonist |  |  |  |  |
|  | naive | 6 | n/a | n/a | n/a |
|  |  |  |  |  |  |
| protein analysis | PBS | 10 | 67.6 ± 1 | 581.6 ± 69.7 | 0 |
|  |  |  |  |  |  |
|  | TNFR2 | 11 | 66.2 ± 1.7 | 557.5 ± 84.8 | 1 |
|  | agonist |  |  |  |  |

* Animals not included in the sample because of inadequate injury (high BMS score on day 1).

Supplementary table 1. Groups and injury parameters used for the study.

| Gene | Forward Primer | Reverse Primer | Product size (bp) |
| --- | --- | --- | --- |
| CXCL10 | 5’-gccgtcattttctgcctcatcct | 5’-ctcattctcactggcccgtcatc | 113 |
| CCL2 | 5’-ccccactcacctgctgctac | 5’-cctgctgctggtgatcctctt | 86 |
| IL-1β | 5’-gtgtctttcccgtggacctt | 5’-cgtcacacaccagcaggtta | 121 |
| IL-33 | 5’-ggtcccgccttgcaaaata | 5’-tcccgtggataggcagagaa | 189 |
| TNF | 5’-aggcactcccccaaaagatg | 5’-tcaccccgaagttcagtagacaga | 123 |

Supplementary table 2. List of primers used for qPCR analysis.
